# Stimuli-responsive microgels for controlled deposition of gold nanoparticles on surfaces

**DOI:** 10.1039/d0na00656d

**Published:** 2020-10-30

**Authors:** Menglian Wei, Wenwen Xu, Feng Gao, Xue Li, Wildemar S. P. Carvalho, Xueji Zhang, Michael J. Serpe

**Affiliations:** Key Laboratory of Optoelectronic Devices and Systems, College of Physics and Optoelectronic Engineering, Shenzhen University Shenzhen 518060 China; Department of Chemistry, University of Alberta 11227 Saskatchewan Drive Edmonton T6G 2G2 Canada michael.serpe@ualberta.ca

## Abstract

A variety of gold nanoparticle (AuNP) core/poly(*N*-isopropylacrylamide) (pNIPAm) shell microgels (Au@pNIPAm) were generated using seed-mediated polymerization. The shell thickness and AuNP core diameter were easily tunable at the time of synthesis. The resultant Au@pNIPAm microgels were characterized *via* photon-correlation spectroscopy, transmission electron microscopy and ultraviolet-visible spectroscopy. AuNP arrays were generated by “painting” the microgels on a surface, using the shell thickness to define the distance between the AuNPs, followed by shell removal *via* plasma etching. We found that when the pNIPAm shell thickness decreased (*via* its tuning at the time of synthesis or deposition at elevated temperature at which the shell is collapsed) the AuNPs were closer to one another. We also showed that *via* sequential deposition Au@pNIPAm microgels with different AuNP core sizes could be deposited on a single surface. The presented “painting protocol” offers a facile way to coat large area surfaces quickly which is not easily achievable using other approaches. We envision that this approach is extremely versatile, allowing a number of different nanomaterials embedded in pNIPAm shells to be deposited/patterned on surfaces. With the control over the deposition on the surface that we show here, we hope that the Au@pNIPAm microgels will find use in lithography/surface patterning applications.

## Abstract 2

A variety of gold nanoparticle (AuNP) core/poly(*N*-isopropylacrylamide) (pNIPAm) shell microgels (Au@pNIPAm) were generated and used to control AuNP deposition on surfaces.

## Graphical abstract

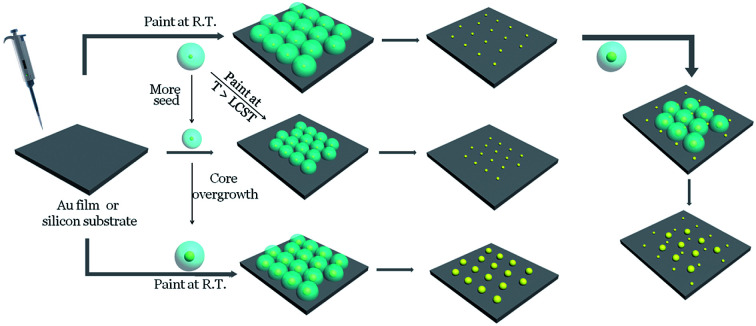

## Introduction

Au nanoparticles (AuNPs) exhibit unique optical properties arising from localized surface plasmon resonance (LSPR) and have been used for various applications, *e.g.*, photonics,^1^ electronics,^2^ catalysis,^3,4^ and sensing/biosensing.^5^ Specifically, ordered arrays of Au nanofeatures (particles,^6^ pillars,^7,8^ and holes^9,10^) on solid substrates have attracted a great deal of attention due to the interesting optical properties as a result of nanoparticle coupling on a surface dictated by the nanomaterial's size, shape and periodicity (among other things).^11^ For example, a surface enhanced Raman scattering (SERS) signal can be significantly enhanced at “hot spots” generated between nanomaterial features.^12–17^ Furthermore, the uniformity of the size and shape of the nanomaterials, and the extent of defect-free periodicity, will dictate the homogeneity of the optical properties.^18^

The semiconductor industry is continuously pursuing new technologies that can generate ever smaller nanoscale features in order to keep up with Moore's law. To this end, a variety of approaches have been applied to fabricate increasingly small nanofeatures, *e.g.*, particles, pillars and holes, using techniques like electron-beam lithography (EBL)^19^ and focused ion beam lithography (FIB).^20^ These techniques provide high resolution and are able to yield features of 1–2 nm with fine control. However, they are made using a serial process, which is inherently low throughput and not ideal for industrial applications. Nanosphere lithography (NSL) has emerged as an alternative method to fabricate ordered nanostructures over a large surface area that is low-cost and scalable.^21,22^ In one example, hard polystyrene (PS) spheres were self-assembled into a hexagonal close packed structure on a substrate and used as a mask for Au deposition onto the substrate.^22^ That is, Au is deposited on the substrate through the interstices between the assembled PS spheres resulting in an ordered array of Au triangles after PS removal from the substrate.^22^ However, the “intertriangle” spacing can only be tuned by varying the size of the PS particles. Furthermore, it is nearly impossible to control the shape of the deposited structure. Other techniques, such as block-copolymer (BCP) assembly-based surface patterning, have been utilized to fabricate patterned metallic nanoscale features on a semiconductor surface.^23–27^ For example, metal ions can be preloaded and brought into close proximity through the coordination interaction with functional groups in a block copolymer. The metallic features can be generated by subsequent galvanic replacement or *in situ* plasma reduction. Using this method, the surface features (metallic array periodicity and shape) are limited by the composition of the block-copolymer as well as their respective molecular weights.

**Scheme 1:**
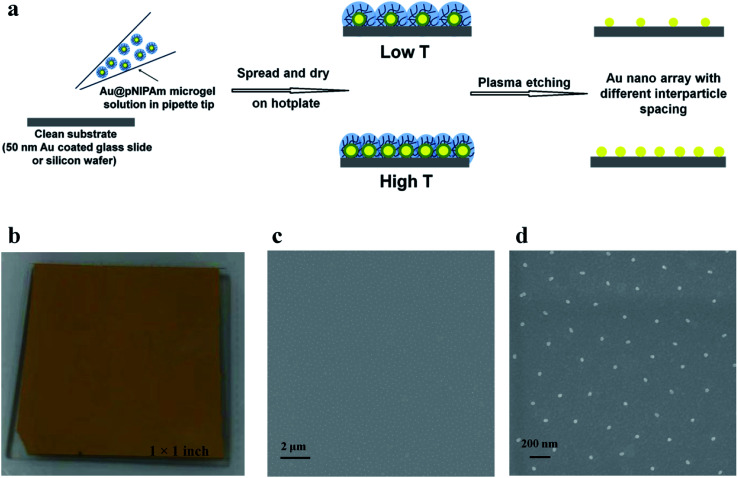
(a) AuNP array fabrication protocol. (b) Images of the Au@pNIPAm microgel coated on a 50 nm Au coated glass substrate. SEM images of a Au@pNIPAm-5 microgel fabricated Au nano array on a silicon wafer with scale bars of 2 μm (c) and 200 nm (d).

Smart polymers have emerged as candidates to replace the PS hard spheres and BCPs for generating metallic nanoscale patterns on surfaces. Smart polymers^28^ (or stimuli-responsive polymers) are capable of undergoing changes in their chemical and/or physical properties upon exposure to external stimuli, such as temperature,^29^ pH,^30^ light,^31^ and analytes.^32^ Poly(*N*-isopropylacrylamide) (pNIPAm) is one of the most well-documented thermoresponsive polymers and exhibits a lower critical solution temperature (LCST) of ∼32 °C.^33^ The pNIPAm polymer chain can undergo a random coil (extended) to globule (collapsed) conformational change as its temperature is raised above the LCST.^33^ In this submission, we establish a novel method for coating large area surfaces with AuNPs of various sizes and center-to-center distances by “painting” AuNP core/pNIPAm shell microgels (Au@pNIPAm) on substrates at varying temperatures. To accomplish this, Au@pNIPAm microgels were synthesized with different pNIPAm shell thicknesses and then self-assembled onto a surface using a previously developed “paint-on” protocol that the group developed.^34^ This approach allows microgels to pack extremely close to one another (*i.e.*, jam pack) on a surface such that their center-to-center distance can be controlled by the diameter of the microgels, *i.e.*, the different shell thicknesses can dictate how close the AuNP cores are to one another. In order to yield bare AuNPs, the pNIPAm shells were removed *via* degradation using plasma etching. Furthermore, the AuNP spacing on the surface can be tuned by depositing the hybrid microgels at temperatures below and above pNIPAm's LCST. That is, at *T* > LCST, the shells are collapsed which allows the Au cores to get closer to one another compared to deposition at low temperature where the shells are swollen. Additionally, the size of the AuNPs in the array can be controlled by overgrowing the AuNP core in the microgel prior to deposition. Finally, we showed that AuNPs with different diameters can be coated on a single surface *via* sequential deposition and etching cycles. Thus, a facile, cost-effective, and highly parallel method of producing AuNP arrays could be achieved with simple control over the spacing between the resulting AuNP features and their size. We acknowledge that while this work is closely related to other work previously reported, we have the ability to very simply coat large areas of substrates very quickly, efficiently, and simply with control over AuNP spacing and the size distribution of the AuNPs on the surface. For example, Clara-Rahola *et al.* reported the generation of 2D ordered Au nanosphere arrays with controllable center-to-center distances by drop-casting AuNP core–pNIPAm shell particles on substrates at different temperature followed by plasma etching.^35^ By taking advantage of the thermoresponsivity of the pNIPAm shell, the array periodicity and optical properties could be altered by controlling deposition temperature. However, in this case, the diameter of the AuNPs in the array was fixed and the tunability of distance between the AuNP features is limited. In another study, Muller *et al.* reported a novel method for creating an array of AuNPs with diameters that vary as a function of spatial position on the surface. This was carried out by pulling a Au@pNIPAm coated surface out of the Au core growth solution containing HAuCl_4_, CTAB and ascorbic acid at a controlled speed.^6^ The size of the Au features is dependent on the amount of time the Au@pNIPAm coated surface is in contact with the growth solution; longer contact led to larger features. This elegant design allows one to produce arrays with multiple Au dimensions. However, the speed of retraction of the slides from the solution has to be carefully programmed such that the desired size or a gradient of Au features in the array is achieved. In this submission, we synthesized a library of Au@pNIPAm microgels with various Au core diameters and shell thicknesses as building blocks to enrich the variety of AuNP arrays. In addition, the “paint-on” protocol allows us to produce uniform AuNP arrays over large surface areas (up to cm^2^ size), as shown in Scheme 1b and c, which is suitable for industrial use. The resultant arrays can potentially be applied in surface-enhanced Raman spectroscopy (SERS) or other nanosensor devices, as well as for computing applications.

## Experimental section

### Materials

Gold chloride trihydrate (HAuCl_4_·3H_2_O) (Aldrich, >99.9%), sodium citrate dihydrate (Aldrich, >99%), sodium dodecyl sulfate (Aldrich, >99%), 3-butenylamine hydrochloride (B-en-A) (Aldrich, >97%), *N*,*N*′-methylenebisacrylamide (BIS) (Aldrich, >99%), acrylic acid (AAc) (>98%), potassium peroxodisulfate (PPS) (Aldrich, >99%), 95% ethanol (Brampton, Ontario), cetyltrimethylammonium chloride (CTAC) (Aldrich, 25 wt%, H_2_O), and l-ascorbic acid (Vc) (BioXtra, >99.0%, crystalline) were used as received. *N*-Isopropylacrylamide (NIPAm) was purchased from TCI (Portland, Oregon) and purified by recrystallization from hexane (ACS reagent grade, EMD, Gibbstown, NJ) before use. Milli-Q deionized water (DI H_2_O) with a resistivity of 18 MΩ cm was used. Glass cover slips were purchased from Fisher Scientific (Ottawa, Ontario). Chromium (99.999%) was purchased from ESPI (Ashland, OR) and Gold (99.99%) from MRCS Canada (Edmonton, AB, Canada). All glassware was cleaned with *aqua regia* and thoroughly rinsed with DI H_2_O.

### AuNP synthesis and functionalization

**Fig. 1:**
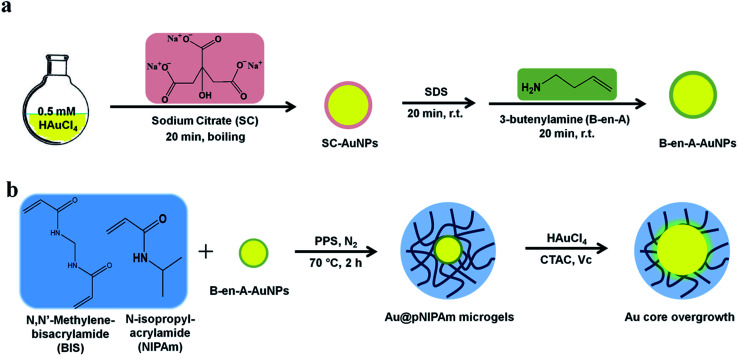
(a) AuNP synthesis procedure. (pink) Sodium citrate is used to generate the (yellow) AuNPs, which are then coated with (green) butenylamine. (b) Au@pNIPAm microgel synthesis and Au core overgrowth procedure. The B-en-A–AuNPs were used as seeds for (blue) pNIPAm shells to grow. Finally, HAuCl_4_ in the presence of CTAC and V_c_ was used to increase the diameter of the AuNP core.

AuNPs were synthesized following the protocol well-established by Turkevich *et al.* as shown in Fig. 1a.^36^ Briefly, a preheated 25 mL sodium citrate dihydrate solution (1 wt%) was added quickly to 500 mL of 0.5 mM HAuCl_4_ with vigorous stirring and strong boiling. After reacting for 20 min, the mixture was allowed to cool to room temperature with slow stirring. AuNPs were then stabilized by adding 3 mL of a 1 mM SDS solution dropwise and stirring for 20 min. Next, 1.63 mL of B-en-A (1.4 mM in ethanol) was added dropwise to the mixture and stirred for another 20 min to generate a hydrophobic surface on the AuNPs. The resultant solution was concentrated by centrifugation at 1100 rcf for 14 h.

### Au@pNIPAm core–shell microgel synthesis

Core–shell Au@pNIPAm microgels were synthesized by seeded precipitation polymerization as shown in Fig. 1b.^37,38^ The microgels' size (or shell thickness) was tuned by varying the Au seed concentration in a fixed amount of monomer solutions. More seeds would generate more nucleation sites, resulting in less monomer polymerization on each AuNP surface and a relatively thin shell compared to when fewer seed particles are added. Experimentally, NIPAm (0.1132 g, 1.000 mmol) and BIS (0.0272 g, 0.176 mmol) were dissolved in 50 mL DI H_2_O and filtered through a 0.2 μm filter into a 100 mL, 3-neck round-bottom flask, which was equipped with a reflux condenser and a temperature probe. The mixtures were degassed *via* N_2_ gas bubbling and heated to 70 °C over 1 h. Next, a AuNP seed solution ([Au] 0.017 M, 50 μL/70 μL/100 μL/200 μL) was added to the heated solution dropwise and stirred continually for 10 min before adding the PPS initiator solution (1 mg in 0.5 mL H_2_O). The red, clear solution became turbid within the first 15 min after initiation of the reaction. The mixtures were allowed to react at 70 °C for 2 h in a N_2_ environment. The dispersions were allowed to cool down to room temperature and filtered through glass wool to remove large aggregates. The microgel solution was cleaned by repeated (6×) centrifugation at ∼10 000 rpm for 30 min followed by resuspension in clean water. The resultant core–shell particles were labeled as shown in Table 1.

**Table 1:** Au@pNIPAm microgel particles synthesized

Sample name	AuNP seed volume (μL)	NIPAm (mmol)	Au@pNIPAm-2 precursor solution (μL)	Feed solution volume (HAuCl_4_ + CTAC) (mL)
Au@pNIPAm-1	50	1.000	—	—
Au@pNIPAm-2	70	1.000	—	—
Au@pNIPAm-3	100	1.000	—	—
Au@pNIPAm-4	200	1.000	—	—
Au@pNIPAm-5	—	—	200	2
Au@pNIPAm-6	—	—	200	5
Au@pNIPAm-7	—	—	200	15
Au@pNIPAm-8	—	—	200	30

### Au core overgrowth

Au core overgrowth was accomplished by reducing HAuCl_4_ in the presence of Au@pNIPAm-2 microgel solutions as shown in Fig. 1b.^39^ First, a 2 wt% Au@pNIPAm microgel solution was diluted with 100 mM CTAC (1 : 1 dilution) to make the precursor solution. In a typical synthesis, a seed solution was prepared by combining 200 μL precursor solutions with 8 mL of 2.4 mM CTAC under vigorous stirring in a 20 mL glass vial, followed by the addition of 130 μL of 10 mM freshly prepared ascorbic acid. Next, a 2 mL feed solution (0.5 mM HAuCl_4_ and 4.75 mM CTAC) was added dropwise to the seed solution while stirring, and the reaction was allowed to proceed for 20 min. The resultant particles were purified by centrifugation at 3740 rcf until the supernatant was colorless, whereupon the supernatant was discarded and the recovered pellet was redispersed in water. This process was repeated twice. The final core size of ∼40 nm was determined by transmission electron microscopy (TEM). Microgels with an overgrown Au core diameter of ∼50 nm, ∼60 nm and ∼75 nm were obtained by adding a 5 mL, 15 mL and 30 mL feed solution, respectively, to the precursor microgel solutions. The ratio of ascorbic acid/HAuCl_4_ was kept constant at 1.3 in all Au core overgrowth experiments.

### Au@pNIPAm microgel assembled film

The Au@pNIPAm microgels were deposited on a 50 nm Au coated glass substrate (1 × 1 inch) or silicon wafer (1 × 1 cm) using a previously reported “paint-on” technique as shown in Scheme 1.^34^ Initially, the 50 nm Au coated glass substrate (or silicon wafer) was cleaned with 95% ethanol and DI H_2_O and dried with N_2_ before use. A 40 μL aliquot of the resultant Au@pNIPAm microgel solution that was preconcentrated *via* centrifugation was dropped onto the pre-cleaned surface and gently spread toward each edge using the side of a micropipette tip. The film was rotated 90 degrees and the solution was again spread to the edges to fully cover the slide. The painting procedure was completed on a hot plate set to a particular temperature. The Au@pNIPAm microgel solution coated substrate was allowed to dry on the hotplate at a fixed temperature for 2 h. Excess microgels not directly adhered to the Au-coated glass substrate were washed away *via* excessive rinsing with DI H_2_O and further soaking in DI H_2_O at 30 °C overnight. As a result of this painting procedure, the microgels are “jam” packed on the Au-coated substrate such that the outer shell surfaces were in contact with each other. We point out again that the spacing between the AuNP cores is controlled by the pNIPAm shell thickness. The same procedure was applied for the silicon wafer except that the volume of the microgel solution was decreased to 10 μL to account for the smaller size.

### AuNP arrays

AuNP arrays were formed by removing the pNIPAm shell from the Au@pNIPAm microgels adhered to the Au-coated substrates by placing the Au@pNIPAm microgel-coated substrates in the chamber of a RIE (Oxford NGP 80) plasma etching system at 100 W in an O_2_ environment. The etching time was varied in order to fully remove the polymer shell without observable degradation of the AuNPs; the AuNPs remained in their original array location. In one example we painted another Au@pNIPAm microgel solution on the etched surface containing AuNPs such that the new Au@pNIPAm microgels can fill in the voids left by the pNIPAm shell etching. We then etched a second time to remove the pNIPAm shell from the newly deposited Au@pNIPAm microgels.

### UV-vis spectroscopy

The absorbance spectra of AuNPs and Au@pNIPAm microgel particles were recorded with an Agilent 8453 UV-vis spectrophotometer, equipped with an 89090A temperature controller and a Peltier heating device. The absorbance spectrum of the Au@pNIPAm microgel particle solution was measured as a function of temperature from 20 to 60 °C in 5 °C increments. The temperature was allowed to stabilize for 5 min before a spectrum was recorded.

### Transmission electron microscopy

Both AuNPs and Au@pNIPAm microgels were characterized with a JEOL TEM instrument (JEM 2100, USA) to investigate the particle morphology and size. The specimens were prepared by drying 10 μL solutions of highly diluted samples on carbon coated copper grids.

### Photon correlation spectroscopy (PCS)

The hydrodynamic diameter of Au@pNIPAm microgel particles was measured by PCS (Brookhaven Instruments ZetaPlus zeta potential analyzer, Holtsville, NY) as a function of temperature from 25 to 60 °C in 5 °C increments. All the measurements were performed in DI H_2_O with an average of ten 30 s acquisitions and an average of three measurements per sample at each temperature.

### Atomic force microscopy (AFM)

The AuNP arrays and Au@pNIPAm microgel film on a 50 nm Au coated glass substrate were imaged by AFM (Digital Instrument, Dimension 3100) in air. The images were acquired using a scan rate of 0.5 Hz and 512 scan points and lines in the tapping mode.

### Scanning electron microscopy (SEM)

The AuNP arrays generated with Au@pNIPAm-5/6/7/8 on a silicon wafer were imaged using a Zeiss Sigma FESEM instrument operated at 5 kV.

## Results and discussion

### Au nanoparticle characterization

**Fig. 2:**
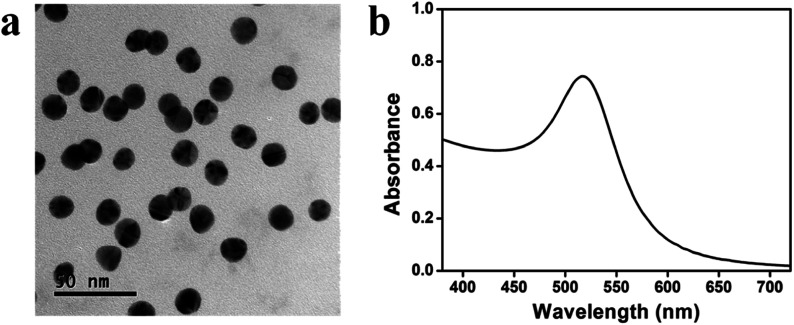
(a) A TEM image of the AuNPs; (b) UV-vis spectrum of a AuNP dispersion in DI H_2_O.

AuNPs were synthesized following the Turkevich protocol and were found to be 14 ± 2 nm (*n* = 156) in diameter, measured by analyzing TEM images using Image J. As can be seen from the TEM image in Fig. 2a, the AuNPs were mostly spherical. Fig. 2b shows the UV-vis spectrum of AuNPs with a prominent absorbance peak at ∼518 nm that is characteristic of AuNPs of this diameter.

### Au@pNIPAm characterization

**Fig. 3:**
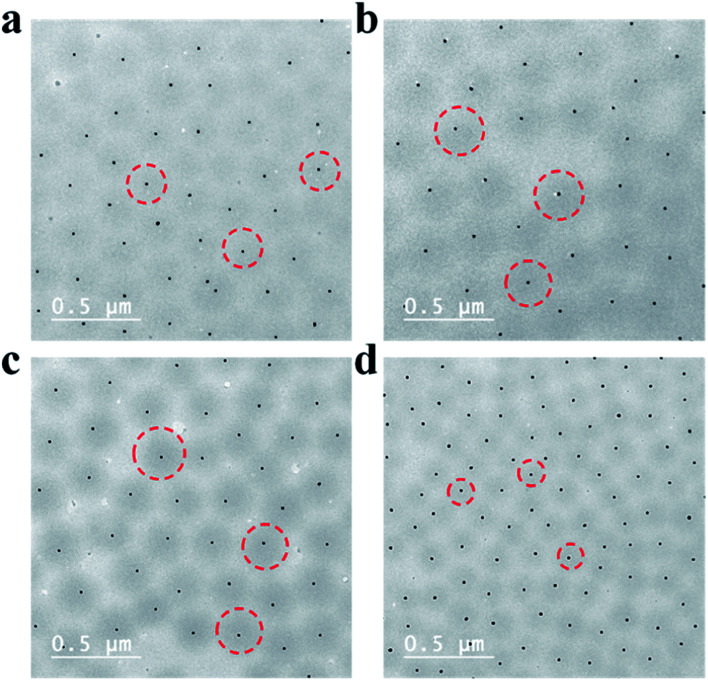
TEM images of (a) Au@pNIPAm-1 microgels, (b) Au@pNIPAm-2 microgels, (c) Au@pNIPAm-3 microgels, and (d) Au@pNIPAm-4 microgels. The red circles in the TEM images are used to outline the pNIPAm shell edge.

**Fig. 4:**
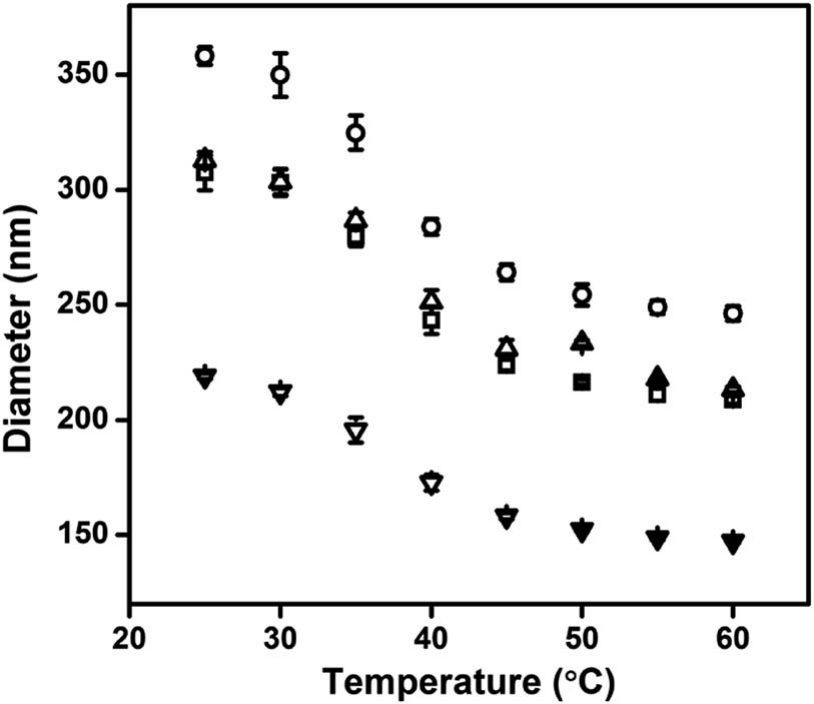
Hydrodynamic diameter of the Au@pNIPAm microgel as a function of temperature, (▢) Au@pNIPAm-1 microgel; (○) Au@pNIPAm-2 microgel; (△) Au@pNIPAm-3 microgel; and (▽) Au@pNIPAm-4 microgel.

Au@pNIPAm microgels with different shell thicknesses were synthesized to generate AuNP arrays with tunable AuNP spacing.^40^ The thicknesses of the shell on the microgels can be tuned by simply varying the AuNP seed to monomer solution concentration ratio. We hypothesized that a thinner shell layer will be formed if more AuNP core “seeds” are used in the reaction owing to the presence of more nucleation sites compared to the case when fewer AuNP core “seeds” are added. The resultant Au@pNIPAm microgel particles with different seed feed ratios were characterized by TEM and are shown in Fig. 3. As can be seen, AuNPs have been encapsulated successfully by the pNIPAm shell in all cases. However, as can be seen in Fig. 3a, 9 particles were formed without a Au core out of a total of 53 when the AuNP seed concentration was low, *e.g.*, 50 μL. In addition, the Au@pNIPAm-1 microgel size was smaller than the size of Au@pNIPAm-2 microgels that were synthesized using 70 μL AuNP seed solution. This can be explained by the fact that the polymerization occurred on the AuNP seeds and in the solution at the same time when the AuNP core concentration was too low, such as for the 50 μL seed solution reaction. When the AuNP seed volume was further increased (*i.e.*, 70 μL to 100 μL and 200 μL), the Au@pNIPAm microgel sizes decreased as we hypothesized, as shown in Fig. 3b–d; neither free AuNPs nor more than one AuNP in one microgel was observed in these TEM images. The thermoresponsivity of the Au@pNIPAm microgels was further investigated *via* PCS by evaluating their size as a function of temperature. As can be seen in Fig. 4, a well-defined volume phase transition could be observed at ∼32 °C for the Au@pNIPAm microgels with different seed feed ratios. This means that the presence of the AuNP core had a negligible effect on the thermoresponsivity of the pNIPAm-based shell. Furthermore, the size of the core–shell particles (or the shell thickness) becomes smaller as the concentration of the AuNP seeds in the reaction increases at each temperature, except for the case with an initial seed volume of 50 μL. The hydrodynamic diameter of these core–shell particles ranged from ∼200 to 350 nm in the solvated state and ∼100 to 200 nm in the collapsed state, which allowed access to a broad shell thickness range.

### Au core overgrowth

**Fig. 5:**
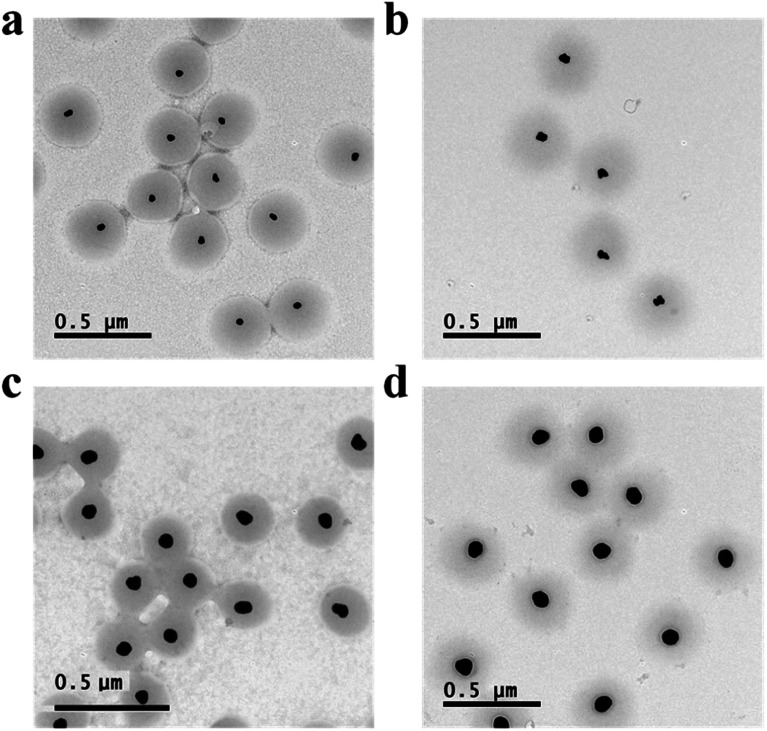
TEM images of Au@pNIPAm microgels with Au core overgrowth. (a) Au@pNIPAm-5, 2 mL feed solution; (b) Au@pNIPAm-6, 5 mL feed solution; (c) Au@pNIPAm-7, 15 mL feed solution; (d) Au@pNIPAm-8, 30 mL feed solution.

**Fig. 6:**
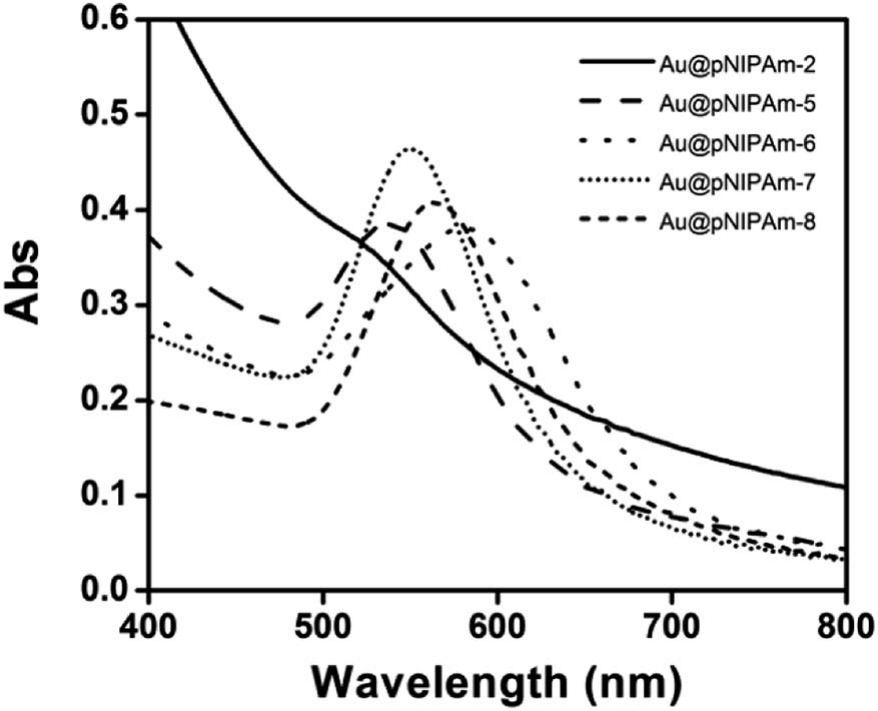
UV-vis spectra of Au@pNIPAm microgels with overgrown Au cores.

To produce arrays composed of AuNPs with different sizes, the AuNP size was varied by further growing the AuNP core in the core–shell microgels by reduction of HAuCl_4_ in the presence of Au@pNIPAm-2 microgels, as shown in Fig. 1b.^39,41^ We point out that using this approach saves many synthetic steps that would normally be required to generate the “library” of Au@pNIPAm microgels used here. The resultant microgels with Au core overgrowth were characterized by TEM, as shown in Fig. 5. As can be seen, the Au core increased in size when more feed solution was added to the same amount of Au@pNIPAm-2 precursor microgel solutions. The diameter of the Au cores was measured by analyzing the TEM images with Image J. The final core sizes were 40 ± 3.9 nm, 49 ± 2.6 nm, 59 ± 4.8 nm and 75 ± 3.8 nm when the volume of the feed solution was 2 mL, 5 mL, 15 mL and 30 mL, respectively. It is noteworthy that the shape of the AuNP core remained mostly spherical in the process of overgrowth except for the case of Au@pNIPAm-6. In the TEM image in Fig. 5b, irregular shapes of AuNPs were observed, and the color of the Au@pNIPAm-6 solution was purple. This is ascribed to the ratio of the capping agent (CTAC) to the AuNP cores, which affects the faceting tendency and growth kinetics.^42^ The resultant core–shell microgels were characterized by UV-vis spectroscopy at room temperature. The results are shown in Fig. 6. As can be seen, the characteristic LSPR peak of Au@pNIPAm-2 is not obvious due to the relatively small diameter of the AuNPs relative to the strong scattering effect of the pNIPAm shell. As the Au core diameter increased, the LSPR peak became noticeable and exhibited a red shift. However, in the case of Au@pNIPAm-6 with 5 mL feed solution overgrowth, the LSPR peak of the AuNPs appeared at an even longer wavelength, ∼580 nm, compared to that of the microgels with the largest Au core dimensions owing to the irregular shape of the AuNPs.

### Au nanoparticle arrays

**Fig. 7:**
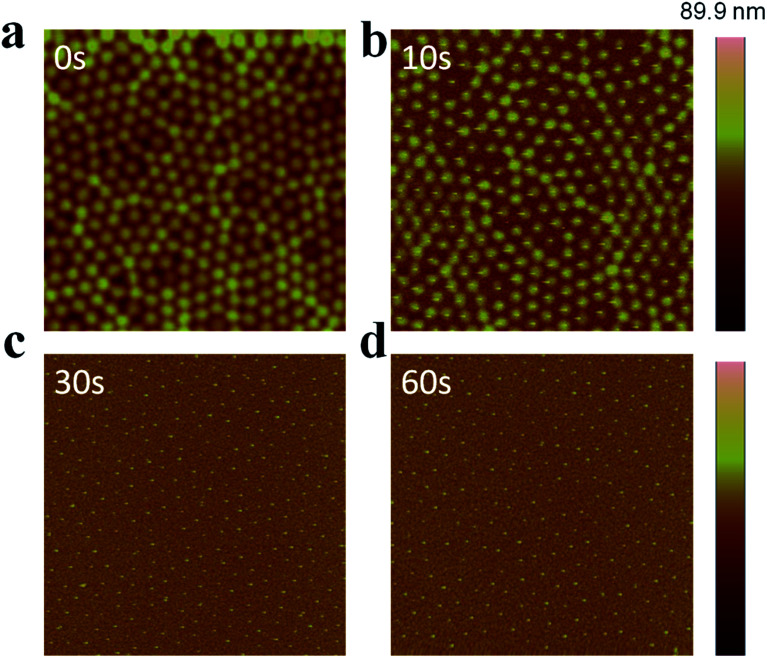
AFM images of Au@pNIPAm-2 microgels deposited at 25 °C after plasma etching for (a) 0 s, (b) 10 s, (c) 30 s, and (d) 60 s. All the scan areas are 5 × 5 μm.

AuNP arrays with tunable and variable interparticle spacing and core dimensions can be generated with the synthesized Au@pNIPAm microgels. To obtain AuNP arrays, the pNIPAm shells were removed from the Au@pNIPAm microgels after painting on the substrates *via* etching in a RIE plasma etching system at 100 W in an O_2_ environment. First, we determined the etching time required to remove the whole pNIPAm shell while not impacting the AuNP core. Specifically, Au@pNIPAm-2 microgel solutions were painted on four 50 nm Au coated glass substrates at 25 °C and further exposed to O_2_ plasma for 0 s, 10 s, 30 s and 60 s, respectively. The resultant slides were characterized by AFM, and the images are shown in Fig. 7. The spherical Au@pNIPAm microgel particles were jam packed on the Au coated glass substrate as a result of the painting procedure before exposure to the plasma. The AuNP cores were not observable by AFM as they were completely covered with the pNIPAm shell. When the film was exposed to plasma for 10 s, the polymer shell degraded, which revealed some of the AuNP core. As can be seen in Fig. 7b, the AuNP cores remained in the center of the Au@pNIPAm microgels during the etching process, which shows that the pNIPAm shell is capable of acting as a mechanical spacer to define the distance between the AuNPs. After a 30 s exposure (Fig. 7c), no polymer shell was observed around the AuNPs and the AuNP cores remained on the surface. Similar results could be obtained for the 60 s exposure. We found that the height difference between the substrate and center of AuNPs in the cross-sectional profile in Fig. 7c and d is comparable to the AuNP diameter measured by TEM in Fig. 2a (data not shown). Therefore, we concluded that a 30 s plasma exposure was sufficient to completely remove the pNIPAm shell from the microgels. That is, AuNP arrays could be obtained after exposure of a Au@pNIPAm microgel film to O_2_ plasma etching for 30 s with no observable effect on the array structure or on the remaining AuNPs.

### Tunability of the interparticle distance of Au nanoparticle arrays

**Fig. 8:**
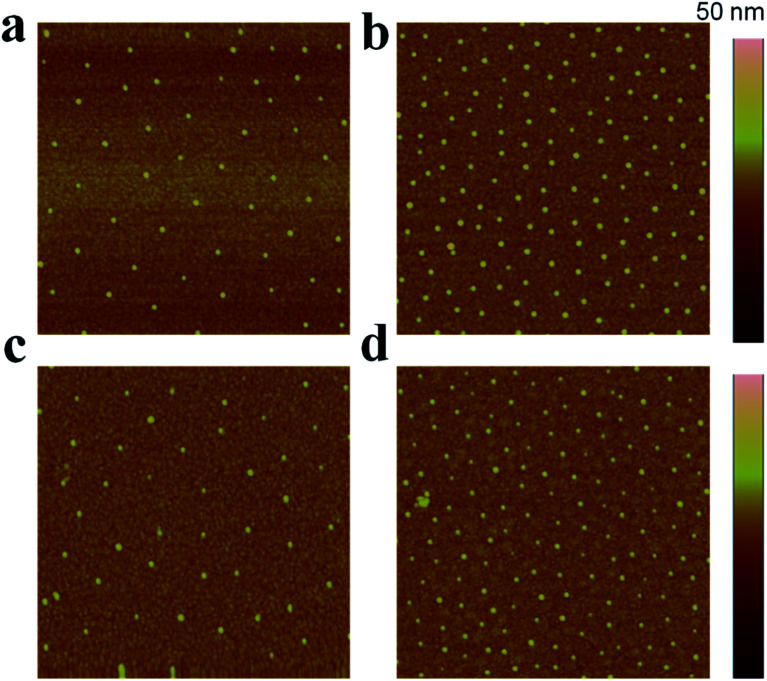
AFM images of the AuNP arrays generated by depositing Au@pNIPAm-2 microgels on a 50 nm Au coated glass substrate at 20 °C (a) and 60 °C (c) followed by shell removal *via* plasma etching. AFM images of the AuNP arrays generated by depositing Au@pNIPAm-4 microgels on a 50 nm Au coated glass substrate at 20 °C (b) and 60 °C (d). All the scan areas are 2 × 2 μm.

**Fig. 9:**
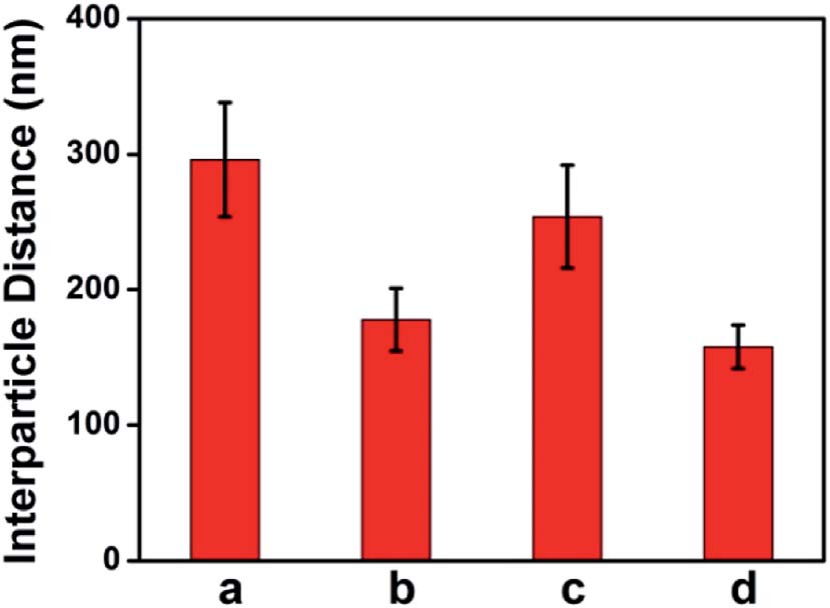
AuNP interparticle distances measured from AFM images. The AuNP array was generated from (a) Au@pNIPAm-2 microgels deposited at 20 °C, (b) Au@pNIPAm-4 microgels deposited at 20 °C, (c) Au@pNIPAm-2 microgels deposited at 60 °C, and (d) Au@pNIPAm-4 microgels deposited at 60 °C.

Due to the manner in which the microgels are deposited on the surface *via* the painting protocol, the interparticle distance between AuNPs in the Au–pNIPAm microgels is ultimately dictated by the thickness of the pNIPAm shell at the time of deposition. Intuitively, such parameters can be easily controlled by painting Au@pNIPAm microgels on a surface with different shell thicknesses. As such, there are two approaches available to generate different shell thicknesses: (1) tuning the AuNPs/monomer ratio during synthesis; (2) varying the temperature at which the painting procedure is completed. To test this, two batches of microgel particles (Au@pNIPAm-2 and Au@pNIPAm-4) with different shell thicknesses were deposited on separate substrates at the same temperature (20 °C). Further, the same batches of microgel particles were painted on other substrates at elevated temperature (60 °C) at which the pNIPAm shells were collapsed. The resultant films were exposed to plasma for 30 s and subsequently imaged by AFM. The results are shown in Fig. 8. As can be seen in Fig. 8a and b, for the depositions at 20 °C larger interparticle distances were obtained for the relatively large Au@pNIPAm-2 microgels (hydrodynamic diameter of 358.2 ± 3.8 nm at 25 °C). The resultant AuNP arrays generated from the Au@pNIPAm-4 microgels (hydrodynamic diameter of 219.1 ± 1.2 nm) exhibit many more microgels in the same scanning area, and closer AuNPs. The distances between the AuNPs in the resultant arrays were measured (100 pairs in total distances each), as shown in Fig. 9. Specifically, the measured interparticle distances were 296 ± 42 nm (column a) and 178 ± 23 nm (column b) for arrays generated from Au@pNIPAm-2 and Au@pNIPAm-4 microgels, respectively. For the deposition at 60 °C, the AuNP arrays generated from both the Au@pNIPAm-2 and Au@pNIPAm-4 microgels exhibited decreased AuNP spacings compared to their deposition at 20 °C. The measured AuNP interparticle distances at 60 °C deposition are shown in Fig. 9. Specifically, the AuNP spacing decreased to ∼254 ± 38 nm (column c) and ∼158 ± 16 nm (column d) for Au@pNIPAm-2 and Au@pNIPAm-4 microgel generated arrays, respectively. In addition, a two sample *t*-test was performed to compare the interparticle distance data from column a and c and column b and d, respectively (100 interparticle distances were compared). Both of the results showed statistical difference at the 99% confidence level. Therefore, the AuNP interparticle spacing can be controlled by either depositing different sizes of microgels at constant temperature or by painting the same batch of microgels at different temperatures. Thus, an AuNP array can be generated with a wide dynamic range of interparticle distances.

### Au nanoparticle array size control

**Fig. 10:**
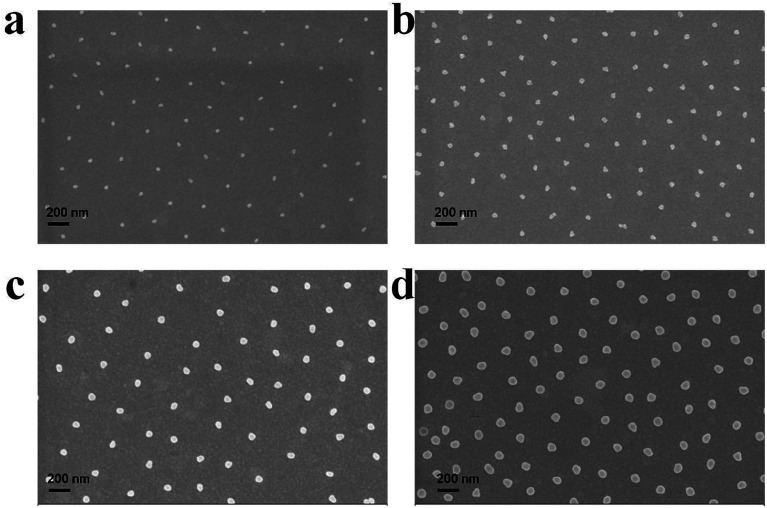
SEM images of AuNP arrays generated from (a) Au@pNIPAm-5, (b) Au@pNIPAm-6, (c) Au@pNIPAm-7 and (d) Au@pNIPAm-8 microgels deposited on a silicon wafer at 25 °C. The scale bar is 200 nm.

The versatility of this approach also allows one to control the size of the AuNPs that comprise the arrays. To do this, overgrown Au@pNIPAm microgels were deposited on a cleaned substrate (silicon wafer, used to facilitate SEM imaging) at 25 °C and the shell was removed by plasma etching. The resultant film was characterized further by SEM, as shown in Fig. 10. From these images, we can see that the AuNPs have the ability to be arrayed on surfaces in hexagonal patterns, dictated by the microgels. Furthermore, we observed the AuNPs to be closer when the AuNP size increased. This could be a result of the shell thickness decreasing as a result of the AuNP overgrowth procedure, although no further studies were completed to understand this phenomenon.

### Arrays with two different Au nanoparticle diameters

**Fig. 11:**
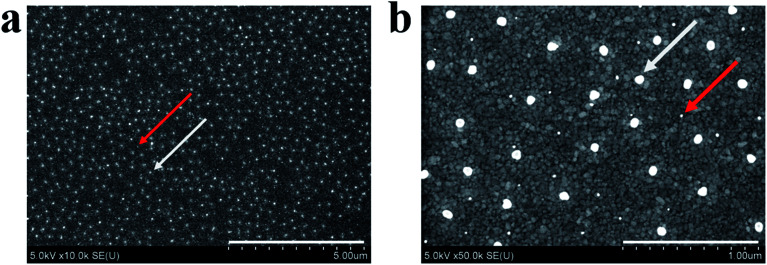
SEM images of the resultant AuNP array achieved *via* consecutive deposition and etching cycles of Au@pNIPAm-2 microgels and Au@pNIPAm-8 microgels. Red arrow: AuNPs from Au@pNIPAm-2 and white arrow: AuNPs from Au@pNIPAm-8. The scale bar is (a) 5 μm and (b) 1 μm.

Finally, we showed that AuNPs with two different diameters can be deposited on a single surface *via* sequential deposition and etching steps. That is, we deposited Au@pNIPAm-2 on a 50 nm Au coated glass substrate at 25 °C *via* the presented painting approach and etched away the pNIPAm shell *via* exposure to O_2_ plasma for 30 s. Subsequently, the Au@pNIPAm-8 microgel solution was painted on the AuNP array at 25 °C and the pNIPAm shell was removed *via* another O_2_ plasma etching procedure for 30 s. The resultant surface was imaged *via* SEM, as shown in Fig. 11, and shows smaller AuNPs (Au@pNIPAm-2) distributed on the surface with larger AuNPs (Au@pNIPAm-8) dispersed in between. We note in Fig. 11a that the distribution of the AuNPs is uniform over a relatively large imaging area, and visual inspection showed that the color of the AuNP-coated surfaces was uniform over the ∼1 cm^2^ substrate surface, which leads us to believe that the surface uniformity is maintained over that surface area. Based on these results, we think that arrays composed of AuNPs with various diameters can be easily assembled on single surfaces utilizing this simple protocol.

## Conclusions

In conclusion, a simple and straightforward approach to generate AuNP arrays was presented that utilizes Au@pNIPAm microgels to control the distance between AuNPs deposited on a surface. We have shown that the distance between the AuNPs on a surface is primarily controlled by the thickness of the pNIPAm shell, which could be tuned at the time of Au@pNIPAm microgel synthesis or by varying the temperature of the Au@pNIPAm microgel deposition. Specifically, when the shell thickness is small, the resultant AuNPs are closer to one another. In addition, we demonstrated that the Au core diameter could be easily tuned using an overgrowth procedure, which allows for added versatility of the approach. Finally, *via* sequential deposition and etching, surfaces coated with different diameters of AuNPs can be easily generated with control over where the AuNPs are deposited on the surface, *i.e.*, they are not randomly distributed on the surface. We think that this approach can be used to control the deposition of a variety of nanomaterials on surfaces, so long as they can be localized in pNIPAm shells. We hope that this approach can find further use in lithography applications, as well as in controlling nanoparticle coupling phenomena, *e.g.*, surface enhanced Raman scattering.

## Conflicts of interest

There are no conflicts to declare.

## Supplementary Material
